# Experimental Investigation of the Deformed Stagger-Jointed Segmental Tunnel Linings Strengthened by Epoxy-Bonded Filament Wound Profiles

**DOI:** 10.3390/ma15196862

**Published:** 2022-10-02

**Authors:** Lei Zhang, Xian Liu

**Affiliations:** College of Civil Engineering, Tongji University, Shanghai 200092, China

**Keywords:** stagger-jointed shield tunnel linings, filament wound profiles, full-scale test, strengthening ling, failure mechanism

## Abstract

A new type of Filament Wound Profiles (FWPs) have been applied to strengthen the deformed stagger-jointed segmental tunnel linings, and a full-scale test was carried out on the ultimate bearing capacity of the linings that are strengthened by the new FWPs. The failure phenomena and the main experimental results were obtained, including the load-displacement curve, strain and bond failure. The internal forces of the FWPs in the strengthened lining were calculated and discussed. The failure chain and weak sections of the strengthened lining were discussed. The overall strengthening benefits were summarized. The results show that: (1) The FWPs were in the state of compression bending or tension bending, and bore part of the axial force and bending moment in the strengthened lining. (2) The initial failure of the strengthened linings was caused by the bond failure between the FWPs and the concrete linings at 0°. (3) The filament wound profiles strengthening method can effectively improve the ultimate bearing capacity and stiffness of the stagger-jointed shield tunnel linings.

## 1. Introduction

The number of tunnel diseases that are found in metro shield tunnel linings are gradually increasing with the dual effects of the natural environment and the service that they provide. Through long-term monitoring and investigation, the diseases of the metro shield tunnel linings include the leakage of water, the cracking and spalling of concrete and the longitudinal settlement and circumferential convergence of the tunnel [1,2,3]. Among them, the excessive circumferential convergence of the tunnel is one of the most important diseases endangering its structural safety, and the large deformation of the tunnel threatens the tunnel clearance. The purpose of shield tunnel strengthening is to improve the rigidity of it to ensure that the linings will not be excessively deformed, improve the bearing capacity and prevent the lining from collapsing.

The internal support is added to improve the overall bearing capacity and rigidity of the tunnel linings with large circumferential convergence, and the materials that are used include fiber-reinforced polymer (FRP) [4], steel plates [5,6,7] and ultra-high-performance concrete (UHPC) [8]. The FRP is pasted on the intrados of the linings to work together with the segments. However, with its one-way tensile property, the FRP can only be applied to the intrados’ tensile zone of the concrete linings. The thickness of FRP is only 0.2 mm, and the steel plate thickness is 20 mm. Therefore, the benefits of the rigidity and bearing capacity improvement of the FRP strengthening method are much lower than that of the steel plates strengthening method. Steel plates have been used in practical projects in Britain [9], Japan [10], Shanghai [11], Taiwan and China [12]. Liu [5] carried out a full-scale test on the deformed continuous-jointed shield tunnels that were strengthened by epoxy-bonded steel plates, and they obtained the conclusion that the failure of the bonding surface between the steel plate and the concrete lining was the key point for the failure of the strengthened linings. The test proved that the steel plates could greatly improve the rigidity and bearing capacity of the deformed tunnel. However, the self-weight of the steel plates is so high that the construction procedure needs to employ hoisting machine whose transportation takes much time, while the steel plates also need to be welded during construction. Therefore, the construction efficiency of the steel plates is insufficient. Liu [8] carried out full-scale tests on UHPC-strengthened continuous-jointed shield tunnel linings. The tests proved that UHPC could improve the rigidity and bearing capacity of the deformed segmental tunnel linings, and the failure of them was controlled by the joints. However, the construction efficiency of the UHPC method is low because the UHPC method requires formwork and long-term curing.

In order to increase the efficiency of the strengthening construction process, Liu [13] proposed a new strengthening method which was denoted as the epoxy-bonded FWP method, and a full-scale study was carried out to test the effect that it had on the continuous-jointed shield tunnel. However, there is no report about the FWP method having strengthening benefits on staggered-jointed shield tunnels, and there is no discussion on the work mechanism of FWPs in tunnel linings.

The basic information of the FWP is summarized in the present paper first, then, the full-scale test is carried out in which a new type of FWP is applied to strengthen the deformed stagger-jointed shield tunnels. Finally, the work mechanism of the FWPs, the failure chain and the weak sections of the strengthened linings are analyzed, and the strengthening benefits are summarized.

## 2. Filament Wound Profiles

FWPs refers to concrete-filled CFRP-steel tube (CFRP-CFST) column members that are used in building structures [14]. Within this, the steel tube is used as the outer reinforcement of the concrete; CFRP can effectively delay the local buckling of the steel tube and improve the bearing capacity and durability. The combination of CFRP and the steel tube can make up for the lack of ductility of concrete. The CFRP-wrapped steel tubes are light in weight, and the non-grouted FWPs can be fixed by a manual operation which means that all of the deformed linings of one tunnel could have the non-grouted FWPs applied to them as internal supports at the same time, and then, the FWPs can be formed after pressure grouting is performed. Therefore, FWP not only makes use of the characteristics of different materials, but also improves the efficiency of the strengthened construction.

**Figure 1:**
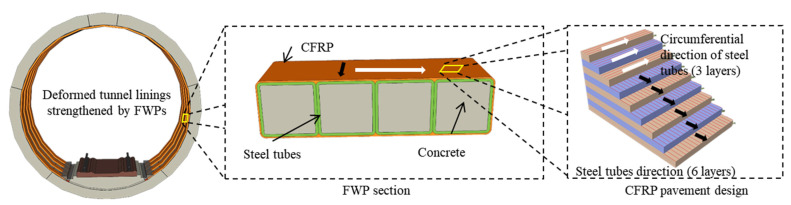
The CFRP pavement design of FWP.

For the FWPs that are used in this full-scale test, the CFRP layer is designed conceptually. As shown in Figure 1, the steel tubes are wrapped with six layers along the steel tube direction and three layers along the circumferential direction. The CFRP along the circumferential direction of the steel tubes can effectively limit the local buckling effect and the bulging deformation of the steel tubes when the FWPs are compressed. The longitudinal CFRP can work together with the steel tubes to bear the tensile stress when the FWPs are subjected to bending or tension. The test was carried out and mechanical properties of the FWPs were obtained [15].

## 3. Full-scale Experiment

### 3.1. Experimental Program

#### 3.1.1. Strengthening Method

**Figure 2:**
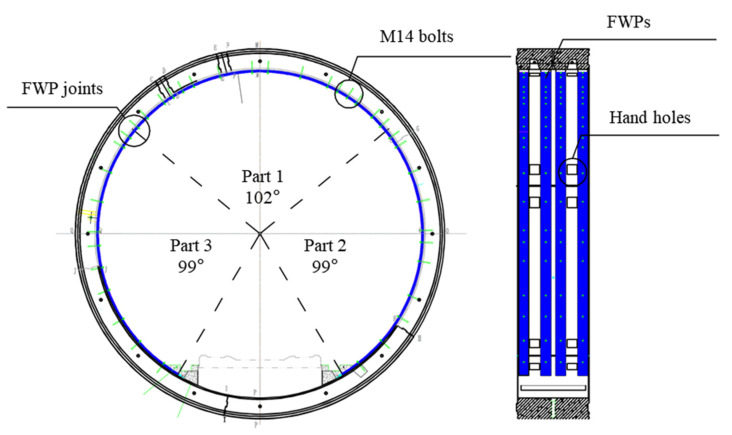
Filament wound profile strengthening method.

The epoxy-bonded FWP strengthening method is illustrated in Figure 2. The FWPs are added to the intrados of the deformed lining. The strengthening components are comprised of three parts. The central angles of parts 1, 2 and 3 are 102°, 99° and 99°, respectively. There are four FWPs which are deployed along the width direction of the tunnel lining, avoiding the hand holes of the segments. The strengthening procedure consists of the following steps: firstly, holes are drilled for someone to place bolts on the segments and the intrados concrete are carved to increase the contact area for the epoxy; secondly, the plug bolts are employed to fix the FWPs onto the surface of the tunnel linings; thirdly, the gap between the FWPs and the tunnel lining is injected with structural adhesive; lastly, concrete is grouted into the cavity of FWPs under a pressure condition.

#### 3.1.2. Experimental Specimen

**Figure 3:**
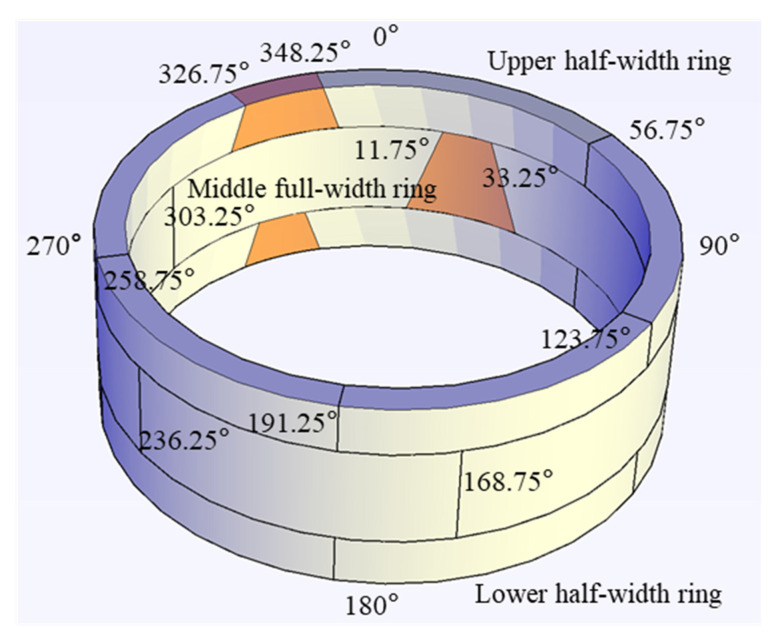
Stagger-jointed segmental tunnel lining specimen.

As shown in the Figure 3, the specimen consists of a full ring and two half-width rings. The key segment of the half-width ring is located at 337.5°, while the key segment of the full-width ring is located at 22.5°. The outer diameter of the full-width ring is 6.2 m, the inner diameter is 5.5 m, the segment thickness is 0.35 m and the ring width is 1.2 m. The full-width ring is comprised of a key segment (K), two adjacent segments (B1 and B2), and three standard segments (A1, A2 and A3), whose central angles are 10.75°, 68.5° and 67.5°, respectively. The concrete grade is C50, and the steel bar grade is HRB335. The adjacent segments are connected with M30 bolts. A tongue and groove structure is used for the longitudinal joints, while a plain joint is used for the circumferential joints.

**Figure 4:**
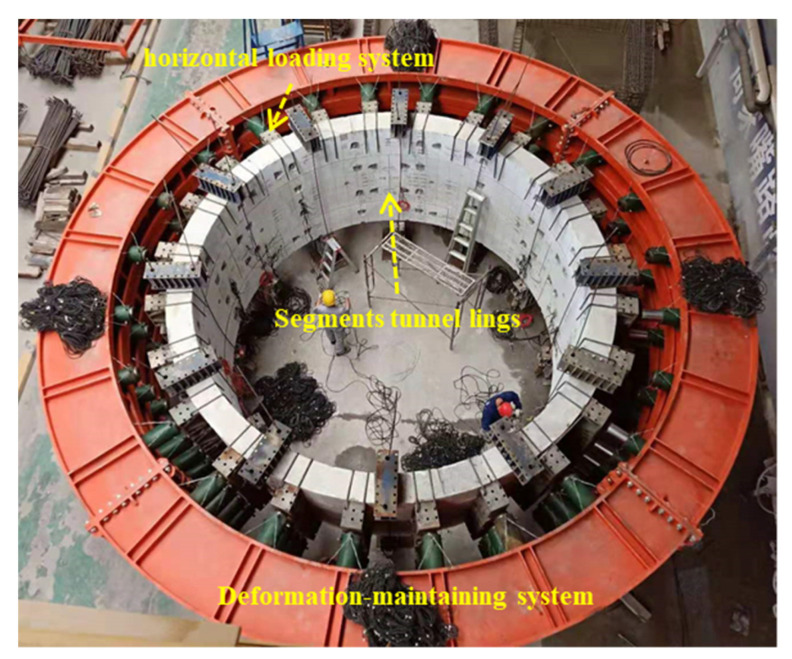
Loading facility.

The loading facility consists of a horizontal loading system, a vertical loading system and a deformation-maintaining system, as shown in Figure 4. The horizontal loading system simulates the water and earth pressure, the strata resistance and the ground overload. It includes 24 loading points, and every loading point has four jacks, four distribution beams and one oil pump. For each jack, the maximal horizontal load is 1000 kN and the maximum displacement is 400 mm. The 24 jacks are controlled by the central host to apply and change the load. During the FWP construction process, the jacks keep working, simulating the earth pressure, while the load fluctuation of the jack is controlled to be within a certain range. The vertical loading system simulates the residual jacking force after the thrust of the shield-tunneling machine. It includes 12 pairs of symmetrical tensile loading points. Each loading points consists of two tensile jacks (200 kN) and two distribution beams. In order to reduce the friction resistance between the segments and the ground that is caused by the self-weight, a steel plate is evenly arranged at the bottom of the whole specimen to ensure the flatness of the ground, and an oil pad is arranged between the bottom of the test specimen and the steel plate to form the sliding support conditions.

#### 3.1.3. Loading Scheme

**Figure 5:**
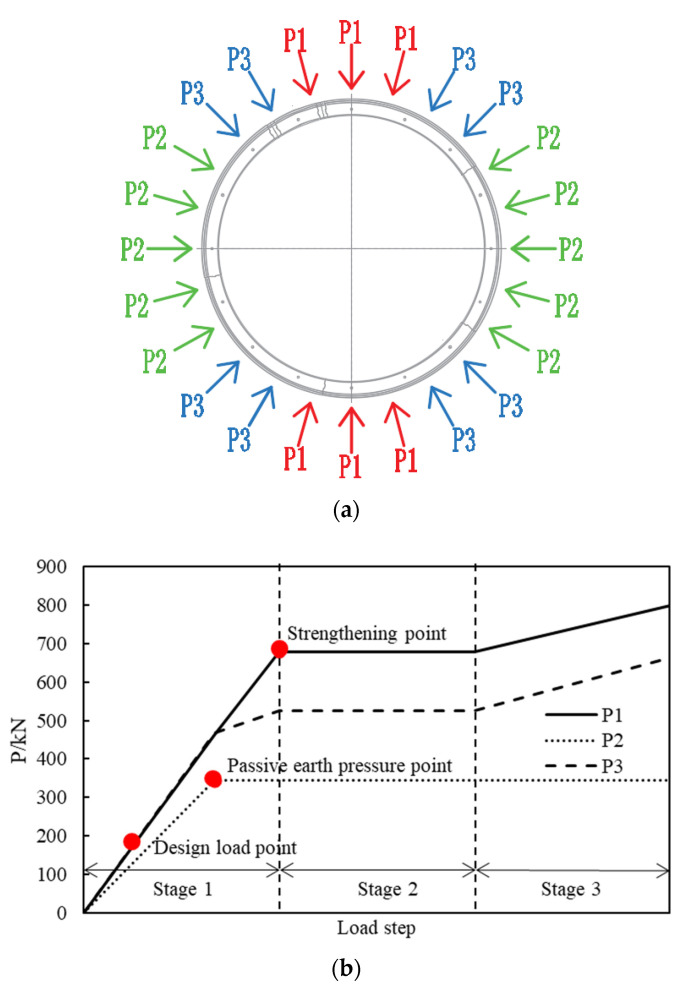
(**a**) Load groups; (**b**) Loading scheme.

The 24 point loads are divided into three groups, as shown in Figure 5a, i.e., six P1s, ten P2s, and eight P3s. P1 simulates the vertical earth pressure, P2 simulates the horizontal earth pressure, and P3 simulates the load on the shoulder of the structure.

Figure 5b illustrates the loading process of the test, which is divided into three stages. In stage one, P1, P2 and P3 increase simultaneously, in which P2 = 0.65 × P1 and P3 = 0.5 × (P1 + P2). When the soil resistance reaches the passive earth pressure, it cannot continue to increase with the increase of the soil displacement and the value of P2 is the product of the passive earth pressure and the area which one jack is responsible for. When P2 reaches the passive earth pressure point, P2 is kept constant while P1 increases continuously, P3 = 0.5 × (P1 + P2). The second stage starts when the structural vertical convergence reaches 83 mm (the strengthening point), the structural deformation is maintained by the deformation maintaining system and the construction of the FWPs is conducted. In stage three, P2 is kept constant, while P1 increases continuously, and P3 = 0.5 × (P1 + P2). This stage ends when the strengthened lining fails.

The following consideration is adopted for the design of the loading scheme: (1) The distribution of the loads that are acting on the linings is similar to the external loading that are acting on the lining in a practical engineering situation. (2) The internal forces of the critical cross section are equal to those in actual tunnel structures that are under operation. The design strategy was employed in the test [5,13,16].

#### 3.1.4. Measurement Program

In the test, the structural deformation, the dilations of the segmental joints, the strains of the steel bars, of the concrete, of the bolts and of the FWP, as well as the relative tangential slippage, and the radial stripping value between the tunnel linings and the FWPs are observed. The summary of the measurement points is listed in Table 1.

### 3.2. Failure State

#### 3.2.1. Structural Failure

When the loads that are acting at P1 reach 677.5 kN, the deformation of the lining is kept constant and the construction of the FWPs is carried out. After the construction is completed, the load scheme is continued. When the loads that are acting at P1 reach 800 kN, the displacement increases rapidly and the overall structural stiffness decreases, wherein this load level is determined as the ultimate bearing capacity load.

**Figure 6:**
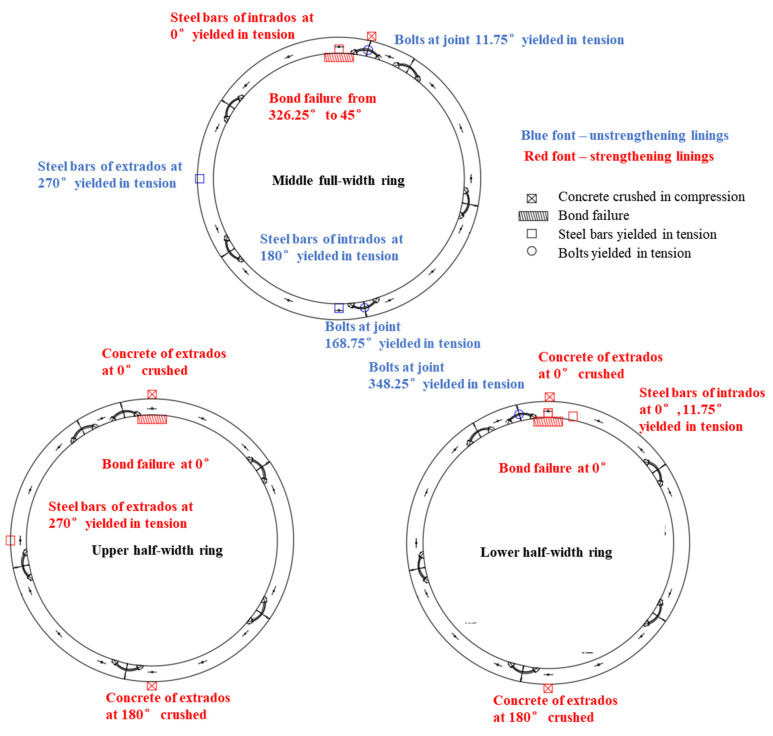
Failure state of the segmental tunnel lining that is strengthened by the FWPs.

The failure state of the strengthened lining is shown in Figure 6. The debonding failure between the FWPs and the segments occurs in the areas ranging from 326.25° to 45°, peeling from both the clockwise and counterclockwise directions. The longitudinal joint at 11.25°of the middle full-width ring is subjected to a sagging moment, while the extrados concrete of this joint is compressed and crushed. The extrados concrete of the upper half-width ring and the lower half-width ring at 0° and 168.75°are crushed because of compression.

There are two kinds of phenomena in concrete segment, one is the tensile cracking of it and the other is the crushing of it under compression. The layout of the cracks and the crush zones is shown in Figure 6c,d. The black solid line represents the cracks, and the shadow represents the position of the crushing zones. The intrados cracks are filled with epoxy resin after the construction of the FWPs, which can improve the durability of the cracked lining.

In the unstrengthened linings, the segments underwent no compression damage, and only tensile cracking occurred. The cracks are on the crown and bottom of the lining intrados, the waist of the lining extrados, and the corresponding positions of the longitudinal joints of the adjacent rings. The maximum width of the crack is near 0° of the lower half-width ring and the maximum width is 0.37 mm. The cracks on the intrados of the middle full-width ring are fully developed, and the average distance between the cracks is about 200 mm.

**Figure 7:**
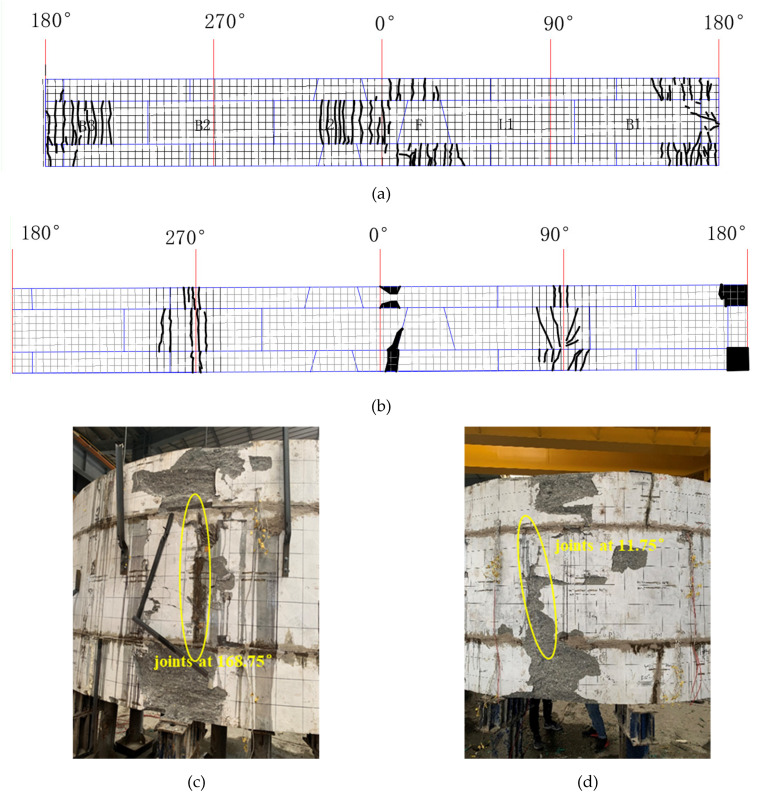
(**a**) Layout of cracks and crushing of intrados concrete; (**b**) layout of cracks and crushing of extrados concrete; (**c**) concrete crushed at 168.75°; (**d**) concrete crushed at 0°.

In the strengthened linings, the cracks on the intrados of the segmental tunnel lining could not be observed because of them being hidden by the FWPs, and the extrados has no new cracks, but only the width of the existing cracks keep increasing. The maximum width of the extrados crack is located at 90° of the lower half-width ring, and the width is 5 mm. As shown in Figure 7a,b, when P1 = 802.5 kN, the extrados concrete at 168.75° of upper and lower half-width rings are crushed by compression forces; whereas when P1 = 835 kN, the extrados concrete at 0° of the upper and lower half-width rings are crushed by compression forces, and the extrados concrete of joint at 11.75°of the middle full-width ring is crushed by compression forces.

**Figure 8:**
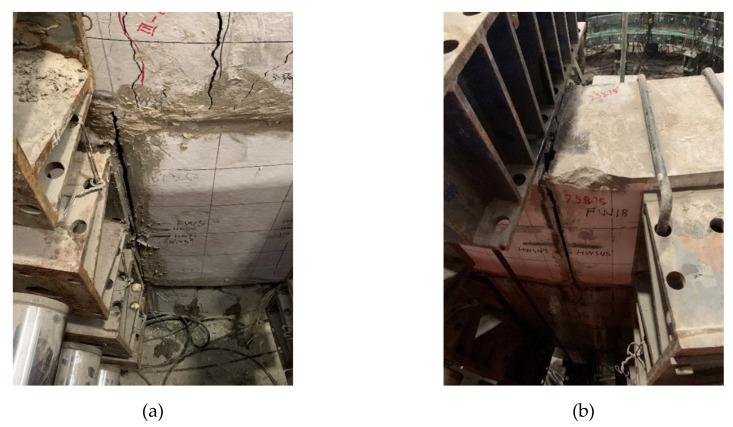
(**a**) Joint 101.25° of full-width ring; (**b**) joint 258.75° of upper half-width ring.

As shown in Figure 8, when the loads that are acting at P1 reach 723.6 kN, the joint at 101.25° of the full-width ring and the joint at 258.75° of the upper half-width ring are under the coupling action of axial forces and hogging moments. Therefore, the intrados of the first joint is compressed, while the extrados of the second joint is pulled, which makes the sealing mortar that is filled into the joints break.

#### 3.2.2. Bond Failure

**Figure 9:**
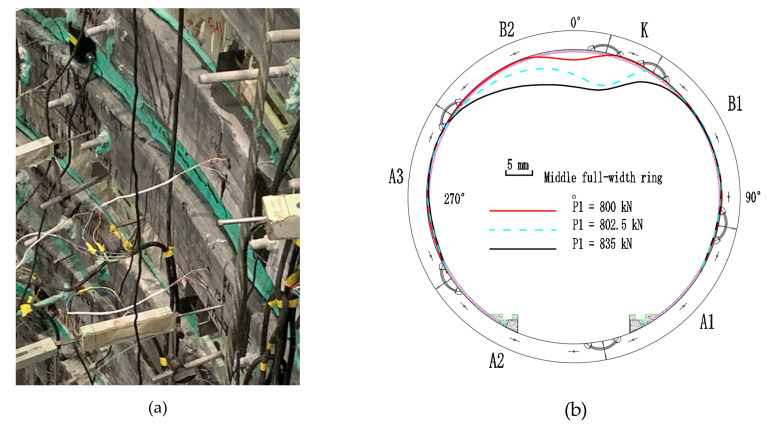

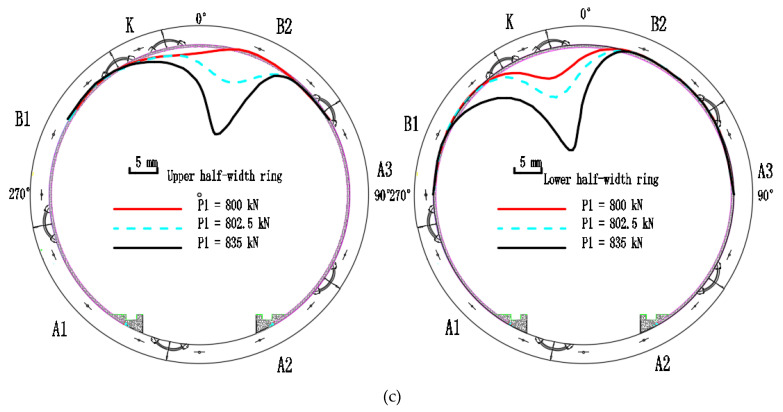
(**a**) Bond failure of middle full-width ring; (**b**) stripping value of middle full-width ring; (**c**) stripping value of half-width ring.

As shown in Figure 9a, when the loads that are acting on P1 reach 800 kN, the bond failure between the FWPs and the full-width ring occurs at 0°. The stripping value of the bond at the failure state is shown in Figure 9b,c.

### 3.3. Experimental Results

#### 3.3.1. Structural Deformation

**Figure 10:**
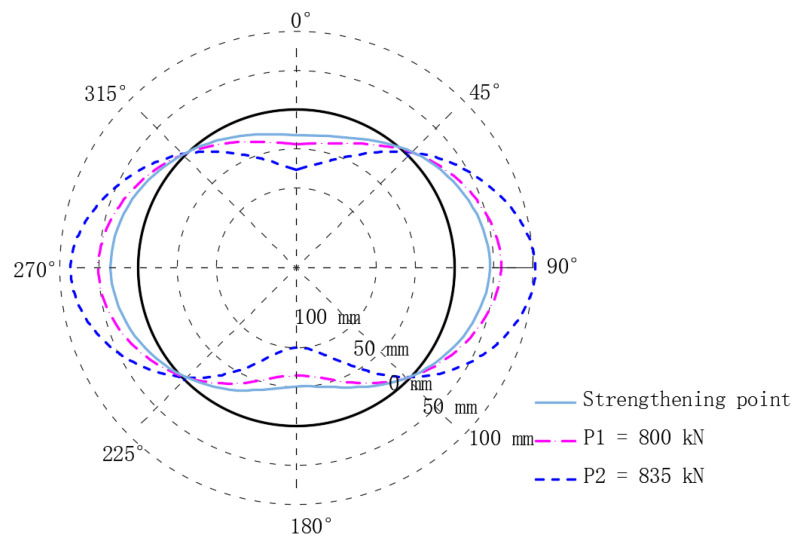
Structural deformation.

As shown in Figure 10, the crown and the bottom of the strengthened linings deform inward, and the waist of the linings deform outward.

At the strengthening point, the vertical convergence is 84.66 mm, and the horizontal convergence is 82.55 mm. When the loads that are acting at P1 reach 800 kN, the vertical and the horizontal convergences are 111.41 mm and 112.37 mm, respectively. When the loads that are acting at P1 reach 835 kN, the vertical and the horizontal convergences are 181.00 mm and 194.13 mm, respectively.

#### 3.3.2. Material Strain

**Figure 11:**
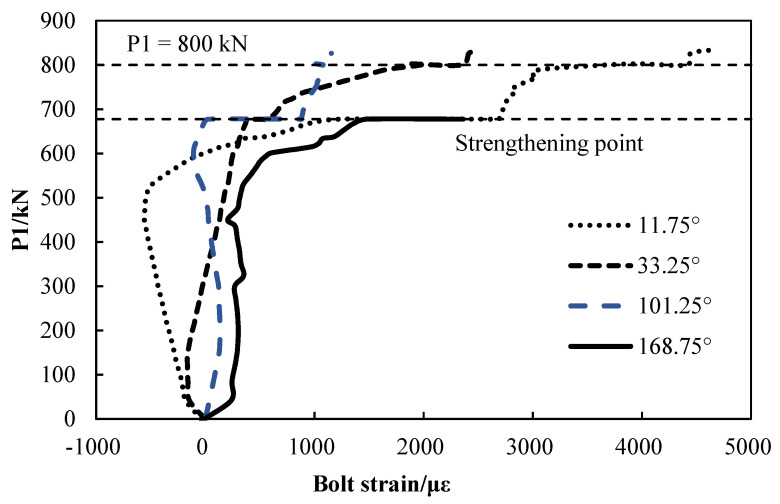
The load–bolt strain diagram.

As shown in Figure 11, the bolts of the joints at 11.75° and 168.75° yield strains that exceeds 2000 με. As the load increasing, the bolt strains at the joint at 33.25° and the joint at 101.25° keep increasing after the FWP strengthening construction procedure is performed. The bolt at the joint at 33.25° yields when the P1 equals 800 kN. The strain gauges of the bolts of the joints at 236.25° and 303.25° in the middle full-width ring are damaged during loading.

**Figure 12:**
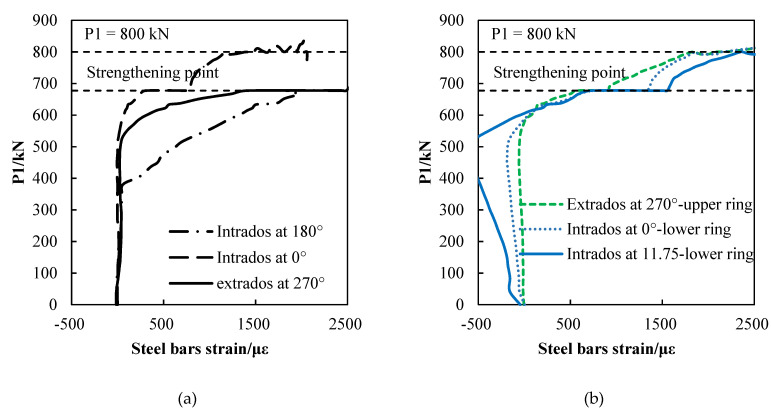
The development of steel bars strains: (**a**) the steel strain of full-width ring; (**b**) the steel strain of half-width rings.

The main reinforcing steel bar of the segment is HRB335, and its elastic strain limit is 2000 με. The crown and the bottom of the linings are subjected to hogging moments, so the intrados steel bars are subjected to tension, and the extrados steel bars are compressed. The waist of the linings, however, is subjected to sagging moments, so the extrados steel bars are subjected to tension and the intrados steel bars are compressed. When the loads that are acting on P1 reach 677.5 kN, the extras steel bars at 270° are tensioned, and the strain of the steel bars rapidly increases to 2000 με, thereby reaching a yielding state. As shown in the Figure 12, when the loads that are acting on P1 reach 800 kN, the strain of the intrados steel bars at 0° increase to 2000 με, thereby reaching a yielding state.

**Figure 13:**
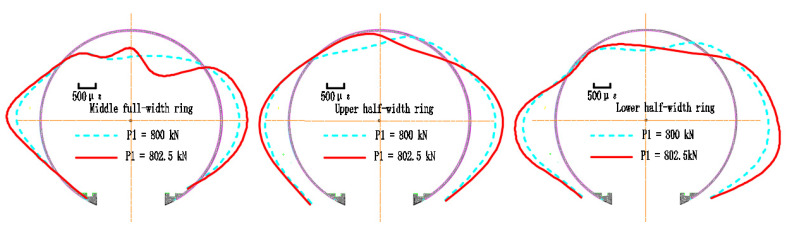
The distribution of FWPs strain.

The strain of the FWP is shown in Figure 13, and the strain gauges measure the surface strain of the CFRP in the FWPs. When the loads that are acting at points P1 reach 800 kN, the distribution of the strain of the FWP is symmetrical along the vertical axis. The FWPs at the crown of the linings are subjected to tensile stress, and the FWPs at the waist are subjected to compressive stress. When the loads that are acting at points P1 are equal to 802.5 kN, the FWPs at 0° have separated from the segment. At the same time, the strain of the FWPs at 0° suddenly decrease, as shown in Figure 13.

As a result of the stripping failure, the superimposition effect between the FWPs at the crown and the segments are weakened. The internal force is redistributed, leading to the strain of the FWPs at the crown decreasing, while the strain of the FWPs at the other positions increases.

**Figure 14:**
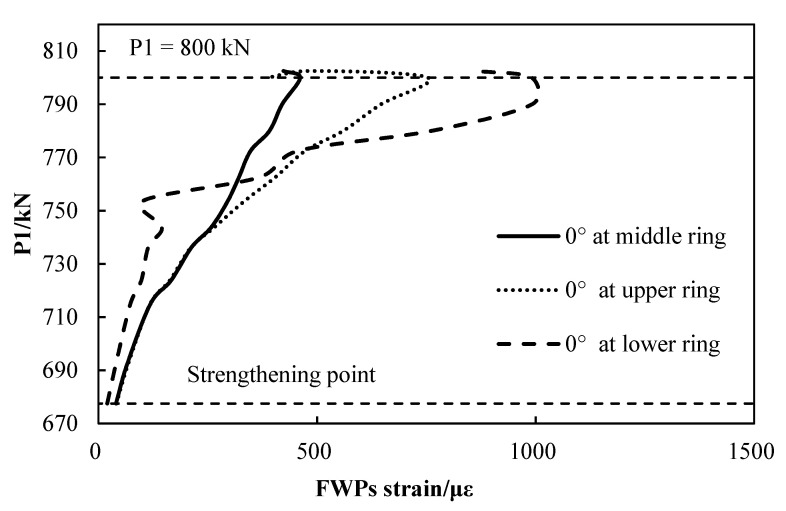
Load-strain curves of FWPs beginning at the strengthening point.

When the loads that are acting at P1 are equal to 800 kN, the strain of the FWPs at 0° has a sudden decrease, which indicates that the bond between the FWPs and the segment fails, as illustrated in Figure 14.

#### 3.3.3. Relative Slip and Stripping Value of the Bond

**Figure 15:**
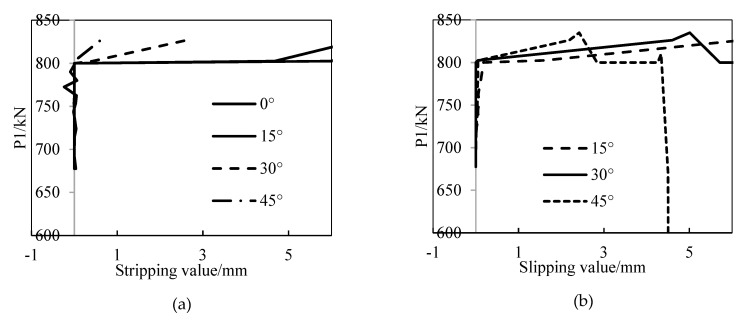

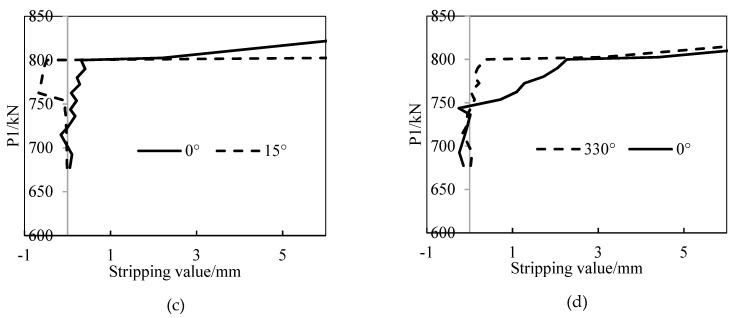
Load-displacement curves of the bond: (**a**) stripping of the middle ring; (**b**) slipping of the middle ring; (**c**) stripping of the upper ring; (**d**) stripping of the lower ring.

The slippage and stripping of the bond between the FWPs and the linings are tested. As shown in Figure 15, the debonding failure happens almost at the same time when the loads that are acting at P1 are equal to 800 kN, which is same as that of the experimental phenomena.

## 4. Discussion

### 4.1. Internal Force of Filament Wound Profiles

#### 4.1.1. Assumption

**Figure 16:**
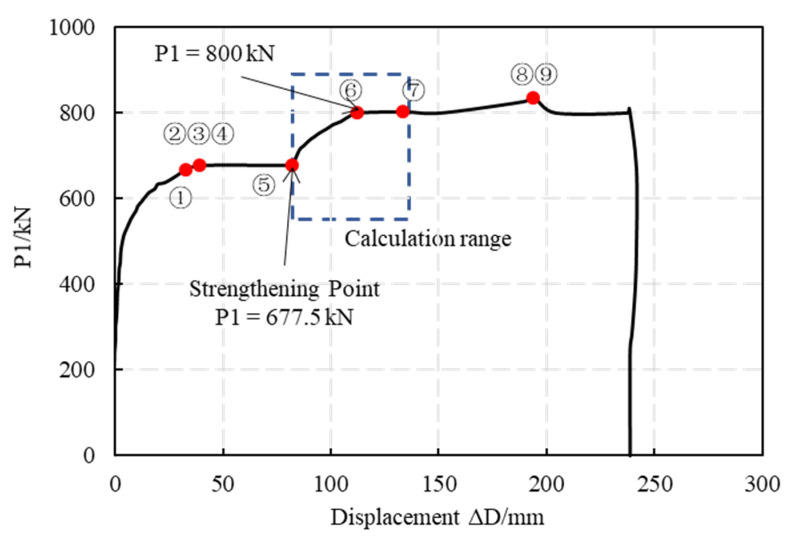
Load-displacement curves for stagger-jointed tunnel lining strengthened by FWPs.

The internal force of the FWPs is calculated in this section and the calculation range is shown in Figure 16. The following assumptions are considered:

(1) Interfacial bonding assumption: The concrete segments and the FWPs are perfectly bonded to form composite linings that bear the external load. When P1 > 800 kN, the bond at 0° between the FWPs and the segments failed, and the strengthened linings at 0° was locally changed from a superimposed structure to a composite structure. With the same boundary and load conditions, the internal force of the composite structure is smaller than that of the superimposed structure [13]. When the calculation is based on a perfect bond, the results is larger than the true internal force of the segments and the FWPs, which is a conservative design.
(2) Material assumptions: The steel conforms to the elastic–plastic assumption, with an elastic modulus of 210 GPa, a yield strength of 420 MPa and an ultimate elongation of 1%. The CFRP is an ideal elastic-plastic material when it is under tension, whose elastic modulus is 235 GPa and ultimate elongation is 1%. The CFRP along the steel tube circumferential direction is not considered, and only the CFRP along the direction of the steel tubes is considered. The concrete is only considered as an elastic–plastic material when it is under compression, whose elastic modulus is 34.5 GPa and ultimate strain is 0.33%.
(1) Plane section assumption: With an incremental load, all of the materials work together while the strain conforms to the assumption of a plane section.

#### 4.1.2. Calculation Results and Analysis

**Figure 17:**
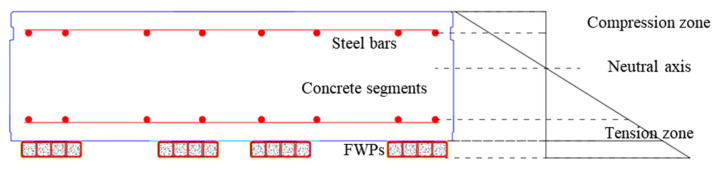
The strain curve of 0°.

For example, as shown in Figure 17, when P1 = 800 kN, the strain curve of 0° is obtained by the linear fitting of the strain of the concrete, steel and FWPs. According to the stress development of the unstrengthened linings, the steel bars at the intrados at 0° have yielded, so the stress of intrados steel bars at 0° keeps yeild strength as loads increasing. The stresses of the concrete and the CFRP are obtained according to the material assumptions that are made. According to the layered strain of each material in the FWPs, the axial force and bending moment of the FWPs can be obtained by integrating the stress. The calculation result is shown in Table 2. The axial force of the total section is −308.14 kN, and the bending moment of the total section is 356.23 kNm. The FWPs bear 471.02 kN tensile force and contribute 103.81 kNm to the bending moment. The FWPs are subjected to an axial tension of 471.02 kN and a bending moment of 0.5 kNm, which means that they are in a tension bending state.

**Figure 18:**
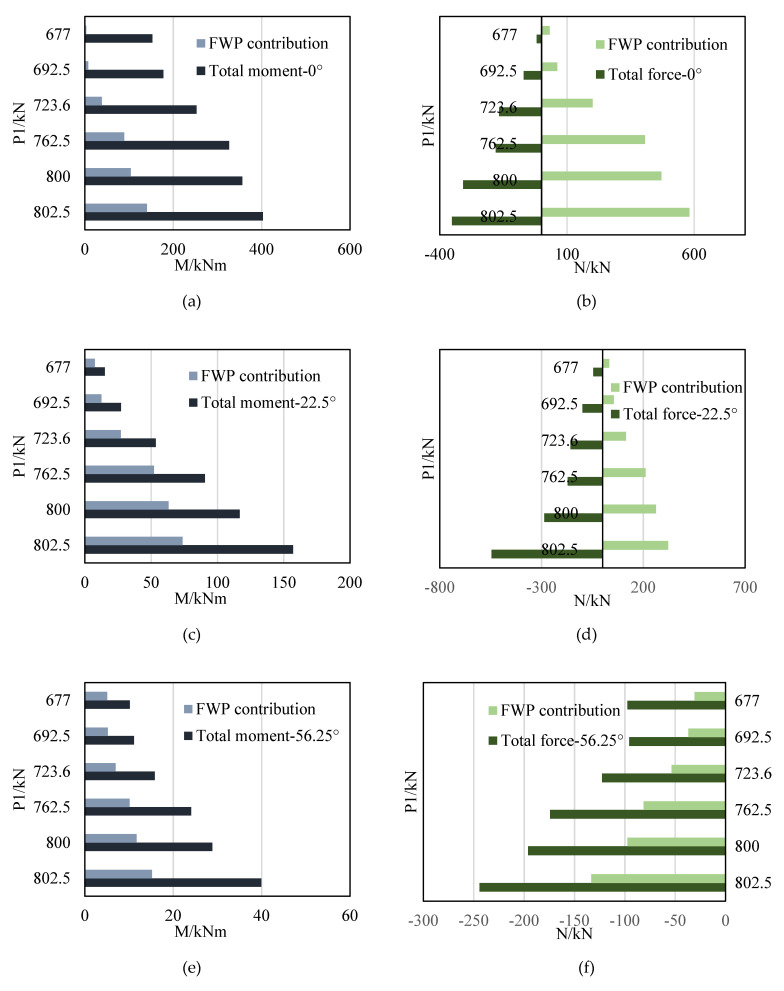

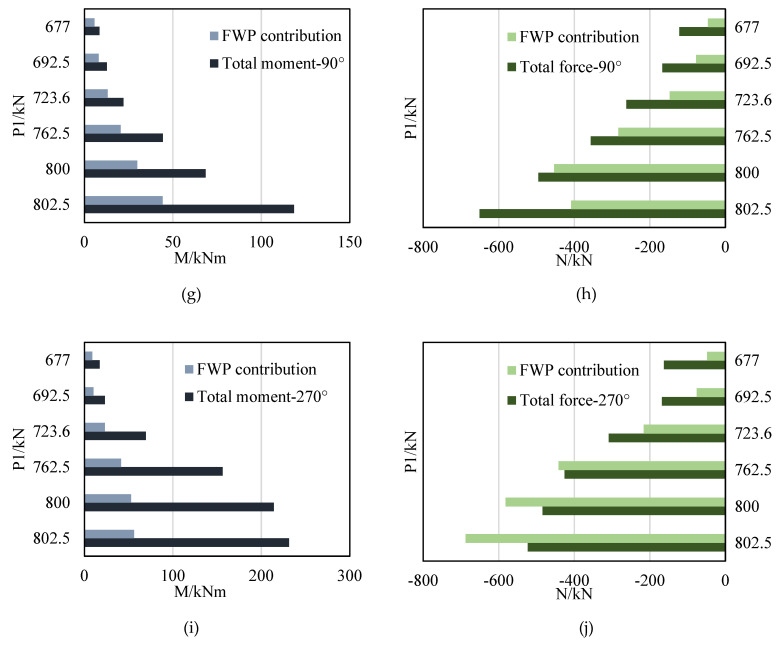
The internal force of segments and FWPs contribution: (**a**) moment at 0°; (**b**) axial force at 0°; (**c**) moment at 22.5°; (**d**) axial force at 22.5°; (**e**) moment at 56.25°; (**f**) axial force at 56.25°; (**g**) moment at 90°; (**h**) axial force at 90°; (**i**) moment at 270°; (**j**) axial force at 270°.

With an incremental load, the internal forces of the segments and the FWPs are shown in Figure 18. The FWPs bear tensile force and bending moments at 0° and 22.5°, while at 56.25°, 90° and 270°, the FWPs bear compressive force and bending moments. With the load increasing, the FWPs and the concrete segments work together in the elastic stage, and the internal forces gradually increase. 

**Figure 19:**
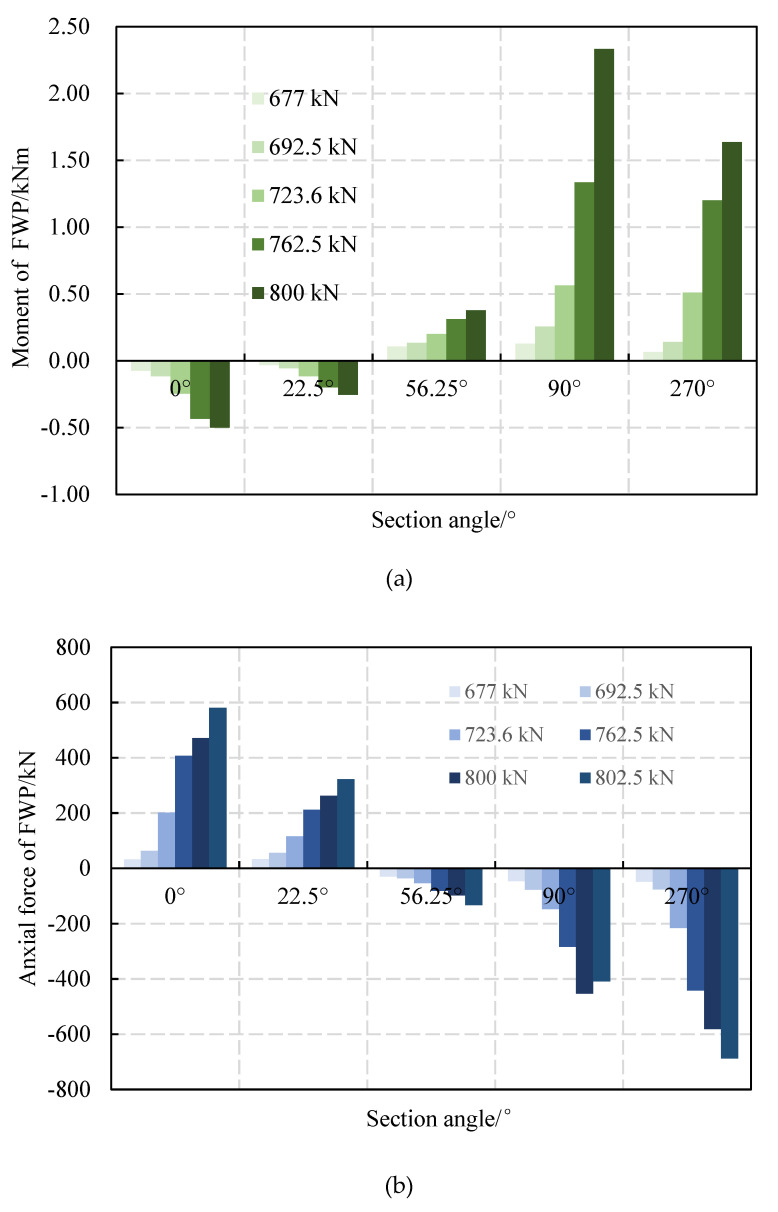
The internal force of FWPs: (**a**) the moments of FWPs; (**b**) the axial force of FWPs.

The internal forces of the FWPs are shown in Figure 19. The FWPs at 0° and 22.5° are in bending and tension states. The FWPs of the 56.25°, 90° and 270° sections are in bending and compression states. When the strengthened linings are in the elastic stage, the internal force of the FWPs increases with the increase of the load. The hogging moment indicates that the surface strain of the FWPs that are not bonded with the segments is greater than that of the FWPs that are bonded with the lining, and the sagging moment indicates the that the opposite is true.

Since the load conditions of the FWP test [15] were axial compression and bending, the discussion about the material utilization rate of the FWPs is limited within the 56.25°, 90° and 270° sections. As shown in Table 3, the utilization rate of the FWPs at 56.25°, 90° and 270° are 8.37%, 41.29% and 47.6%, respectively. Therefore, the FWPs are still in elastic states, without a failure occurring.

### 4.2. Failure Process of Strengthened Segmental Tunnel Linings

#### 4.2.1. Failure Chain

The experimental load-displacement curves of the deformed stagger-jointed segmental tunnel linings that are strengthened by the epoxy-bonded filament wound profiles is illustrated in Figure 16, and the load levels of P1, which are associated with the progressive failure of the specimen, are listed in Table 4.

When the loads that are acting on P1 reach 667.5 kN, the steel bars of the extrados at 270° and the steel bars of the intrados at 180° yield because of the tension that is occurring. When the loads that are acting on P1 reach 677.5 kN, the bolts at the 11.75°and 168.75° joints yield. Therefore, four plastic hinges form in the middle full-width ring at P1 = 677.5 kN, i.e., the 180° section, the 270° section, the joint at 11.75° and the joint at 168.75°. The unstrengthened ring becomes a multi-hinge structure with displacements that are rapidly increasing in the range of small load fluctuations.

The failure process of the linings that are strengthened by the FWPs can be divided into two stages, i.e., the elastic stage and the plastic stage.

When the loads that are acting on the P1 points reach 800 kN, a bond failure occurs between the FWPs and linings from 326.25° to 45°. The superposition between the FWPs and the linings in the crown is ineffective, and internal force redistribution occurs in the structure. The load-displacement curve shows that the structure enters into a plastic state. Its stiffness decreases significantly, with the displacement increasing sharply. This load is defined as the ultimate bearing capacity of the strengthened stagger-jointed segmental tunnel lining.

When the loads that are acting on the P1 points reach 802.5 kN, the extrados concrete of the upper and lower half-width ring at 168.75° is crushed. When the loads that are acting on the P1 points reach 835 kN, the extrados concrete of upper half-ring is crushed by compression at 0°. At the same time, the bolts at the joint at 33.25° yield. Then the loads keep decreasing, and the displacement increases continuously. Then the test is finished.

#### 4.2.2. Weak Sections

The full-scale experiment results indicate that the initial failure of the strengthened staggered-jointed segmental tunnel linings originates from the debonding in the crown, which is same as with the continuous-jointed segmental tunnel lining experiment [13].

The bonding interface is mainly connected by glue, and it is resistant to pull-out and shear. In the sagging moment area of the lining, i.e., the crown and the bottom of the lining, the bonding interface, is in the unfavorable state of shear and tension which makes it easier for interface damage to occur. 

According to the research [7], the peel stress of the bonding interface could be calculated. For example, when P1 = 800 KN, the average tensile stress of the FWPs at 0° is 73.59 MPa, and the inner diameter of the segment and the height of the FWPs are 2.75 m and 0.04 m, respectively. The calculation result of the peel stress of the bonding interface is 4.28 MPa, which is greater than the tensile bearing capacity of the bonding interface, which is 1.5 MPa. Therefore, the bonding interface at 0° is peeled off. Hence, the bonding interface at the crown and the bottom of linings are the weak sections of the FWP-strengthened linings.

**Figure 20:**
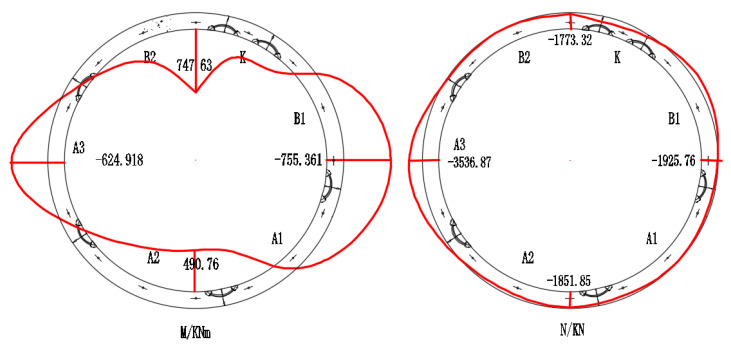
The internal force distribution of unstrengthened middle full-width ring.

The steel bars of the intrados at the crown and bottom of the lining and the steel bars of extrados at the waist are prone to yield failure. The internal force distribution of the unstrengthened lining is shown in Figure 20. For the calculation method and the assumption of the internal force, refer to Section 4.1.1. As shown in Figure 20, the peak values of the sagging moment are at the crown and the bottom of the lining, while the peak value of the hogging moment is at the waist. At this load level, the steel bars of the intrados at the crown and the bottom bear the tensile stress, and the steel bars of the extrados at the waist bear the tensile stress.

The internal force of the strengthened lining with the incremental loads are shown in Table 5. The sagging moment at the crown and the bottom of the lining and the hogging moment at the waist continue to increase. Therefore, the tensile strain of the steel bars at the intrados of the crown and the bottom and the steel bars at the extrados of the waist continue to increase, which are areas that are prone to yield failure. As shown in Figure 12, the steel bars at the 0° zone of the intrados in the middle full-width ring and the steel bars at the 270° zone if the intrados in the middle full-width ring yielded, while the strengthening point and the tensile strain of the steel bars at the 180° zone of the intrados continued to increase after the FWPs construction. Therefore, the steel bars at the intrados of the crown and the bottom of the linings and the steel bars at the extrados of the waist are the weak sections of the FWP-strengthened lining.

After strengthening, the extrados concrete of the joints near the crown and the bottom of the lining is crushed. As shown in Figure 7a,b, the sagging moment is large enough to make the bolts yield under tension and the extrados concrete of the joint crush. After the bonding between the segments and the FWP is intensified, the initial failure of the strengthened lining changed from the bonding failure at the crown to the crush of the 352° zone of the extrados surface joint concrete [17]. Therefore, the extrados concrete of the joints near the crown and the bottom of the tunnel are the weak sections of the FWP-reinforced lining.

In conclusion, the bonding interface at the crown and the bottom of the linings, the steel bars at intrados of the crown and the bottom and extrados of the waist, and the concrete on the extrados of the crown and the bottom joints are the weak sections of the FWP-strengthened linings.

### 4.3. Strengthening Benefits

The FWP strengthening method can effectively increase the ultimate bearing capacity and overall stiffness of the tunnel lining, as shown in Table 6.

The load and waist convergence of the linings at the strengthening point are recorded as *P_0_* and *y_o_*, respectively, while those of the strengthened linings at the elastic limit are denoted as *P_e_* and *y_e_*, respectively. In Figure 16, the slope of the line connecting the strengthening point and point ⑥ represents the rigidity *K_e_* of the strengthened linings. The stiffness of the unreinforced structure is *K_0_*. The ductility is denoted as ∆*y*, given that ∆*y = y_e_ − y_o_*.

The relative increase of the ultimate bearing capacity of the structure *R_p_* and of its overall stiffness *R_k_* are used as criteria for assessing the effectiveness of the FWP strengthening method. *R_p_* is given as *R_p_* = (*P_e_* − *P_0_*)/*P_0_*, and *R_k_* is given as *R_k_* = (*K_e_ − K_0_*)/*K_0_*.

As shown in Table 5, the increase in the ultimate bearing capacity is 122.5 kN and the relative increase is 18.08%. The overall stiffness is 4.11 KN/mm, and the relative increase is 20.55.

## 5. Conclusions

In the present paper, a full-scale test of FWP-strengthened stagger-jointed tunnel linings was conducted. The FWP working mechanism, the failure mechanism of the strengthening linings and the strengthening benefits were analyzed and summarized. The following conclusions are drawn from the experimental investigation:

(1) The FWPs bear the axial force and the bending moment with incremental loads. The FWPs are under tension and a bending state at 0° and 22.5°, while they under in compression and a bending state at 56.25°, 90° and 270°.
(2) Based on the experimental phenomena and the theoretical analysis, the weak sections of the FWP-strengthened linings are summarized. After strengthening, the bond between the crown and the bottom of the lining is prone to bond failure, the extrados concrete of the joint near the crown and the bottom is prone to be crushed, and the steel bars at the intrados of the crown and the bottom and extrados of the waist are prone to yielding.
(3) The stiffness and ultimate bearing capacity of the structure improved significantly with the FWPs. The increase in the ultimate bearing capacity of the stagger-jointed segmental tunnel lining that was strengthening by the FWPs was 122.5 kN, and the relative increase was 18.08%. The overall stiffness of the strengthening structure was 4.11 KN/mm, and the relative increase of stiffness was 20.55.

The results and experimental data that were obtained in this research can be used to make foundation for further investigation into the numerical model of stagger-jointed segmental tunnel linings that are strengthened by FWPs. Subsequently, the internal force of the FWPs will be obtained by calibrating the numerical model to guide the design of the FWP strengthening method.

## Figures and Tables

**Table 1:** Summary of measurement points.

Test Item	Sensor	Range	Precision	Number
Overall deformation	Displacement meter	500 mm	0.01 mm	14
Strain of steel bar	Strain gauge	20,000 με	1 με	416
Strain of bolt	Strain gauge	20,000 με	1 με	48
Strain of concrete	Strain gauge	20,000 με	1 με	308
Joint dilation	Displacement meter	100 mm	0.01 mm	48
Strain of steel FWP	Strain gauge	20,000 με	1 με	308
Relative slippage	Displacement meter	100 mm	0.01 mm	38
Relative stripping value	Displacement meter	100 mm	0.01 mm	38

**Table 2:** The calculation results of internal force at 0°.

Incremental Load	Total Load	The Internal Force of 0°		FWP Contribution		The Internal Force of FWP	
ΔP1/kN	P1/kN	M/kNm	N/kN	M/kNm	N/kN	M/kNm	N/kN
122.5	800	356.23	−308.14	103.81	471.02	0.5	471.02

**Table 3:** The material utilization rate of FWPs.

Section Angle	N/kN	N/Nu	M/kNm	M/Mu	N/Nu + M/Mu
56.25°	−97	6.86%	0.38	1.51%	8.37%
90°	−453	32.04%	2.33	9.25%	41.29%
270°	−581	41.09%	1.64	6.51%	47.60%

**Table 4:** Failure process of strengthened stagger-jointed tunnel lining.

	Load/kN	Displacement/mm	Phenomenon
①	667.5	39.73	the steel bars of intrados at 180° yield because of tension
②	677.5	45.77	the steel bars of extrados at 270° yield because of tension
③			the bolts at joint 11.75° of middle full-width ring yield
④			the bolts at joint 168.75° of middle full-width ring yield
⑤			Strengthening point
⑥	800	112.37	bond failure occurs at 0°
⑦	802.5	133.79	the concrete of extrados at 168.75° is crushed in the half-width ring
⑧	835	194.13	the concrete of extrados at the 0° is crushed in the half-width ring; the bolts at joint 33.25° of middle full-width ring yield

**Table 5:** The internal force of strengthened lining.

	Strengthening Point P1 = 677.5 kN		ΔP1 = 122.5 kN P1 = 800 kN	
Section Angle	M/kNm	N/kN	ΔM/kNm	ΔN/kN
0°	747.63	−1773.32	356.22	−308.14
90°	−755.36	−1925.76	−118.49	−650.44
180°	490.75	−1851.85	Strain gauge breakdown	
270°	−624.91	−3536.87	−214.22	−484.01

**Table 6:** Strengthening benefits of FWPs.

Symbol	Value
$P_{0}$ (kN)	677.5
$y_{0}$ (mm)	82.55
$P_{e}$ (kN)	800
$y_{e}$ (mm)	112.37
$K_{e}$ (kN/mm)	4.11
$K_{0}$ (kN/mm)	0.20
Δy (mm)	29.82
Δp (kN)	122.50
$R_{p}$	18.08%
$R_{k}$	20.55

## Data Availability

Not applicable.
